# Diverging Decisions? A Comparison of Jury Verdict Procedures

**DOI:** 10.3390/bs15121666

**Published:** 2025-12-03

**Authors:** Kayla A. Burd, Valerie P. Hans, Hannah J. Phalen, Stephanie Madon, Max Guyll, Krystia Reed

**Affiliations:** 1 Department of Psychology, University of Wyoming, Laramie, WY 82071, USA; hphalen@uwyo.edu; 2 Cornell Law School, Cornell University, Ithaca, NY 14853, USA; valerie.hans@cornell.edu; 3 School of Interdisciplinary Forensics, Arizona State University, Glendale, AZ 85306, USA; madon@asu.edu (S.M.); guyll@asu.edu (M.G.); 4 Department of Psychology, University of Texas at El Paso, El Paso, TX 79968, USA; kmreed2@utep.edu

**Keywords:** juror decision making, verdict procedures, civil law, race

## Abstract

Courts have the capacity to structure juries’ decision making through the use of general verdicts with answers to written questions, special verdicts, and, in some countries, the requirement that juries provide reasons for their verdicts. Little empirical research has explored the impact of special verdicts or a reasoning requirement on juror decision making in comparison to other verdict procedures. In Experiment 1, mock jurors (*N* = 267) read a summary detailing a case of alleged defamation of a White or Black plaintiff and rendered a verdict (general verdict vs. special verdict vs. two variations of a general verdict procedure with a reasoning requirement). In Experiment 2, mock jurors (*N* = 292) heard a mock trial while viewing photos of a White or Latinx plaintiff and rendered a verdict (general verdict vs. special verdict with a reasoning requirement) after deliberating as a group. In Experiments 1 and 2, mock jurors who rendered a verdict using a general verdict form were more likely to find for the plaintiff compared to those using a special verdict form (Experiment 1) or a modified special verdict form with reason-giving (Experiment 2).

*“Most legal cases that are litigated and appealed are [complex], in that the facts can be ambiguous, incomplete, and contradictory; different rules, values, and principles can be invoked to support opposite conclusions; and the case at hand can be somewhat analogous to more than one previous decision. On their face, such tasks might seem intractable.”*

(Simon, 2004, p. 516)

## 1. Introduction

Jurors are tasked with remembering, integrating, and utilizing large amounts of complex evidence. The above account draws attention to just how difficult it can be for jurors to reach a verdict during trial. Legal and psycholegal scholars have long questioned how jurors make decisions, what evidence and information affect their decisions, and what can be done to improve juror decision making accuracy. Many theories of reasoning describe the complexities of the decision making process generally (for a review, see Osman, 2004), with some paying special attention to decision making in the context of juries (e.g., Pennington & Hastie, 1986, 1988, 1992).

The courts try to promote strong decision making in jurors, and some jury systems have adopted verdict procedures that aim to reduce arbitrary decision making and facilitate judicial review (Thaman, 2011). Some European countries seem especially keen on scaffolding jurors’ decision making (Marder & Hans, 2015), despite the fact that such procedures undermine jury independence (Burd & Hans, 2018). For example, Austria and Spain require that jurors provide substantiated reasons in support of their verdict decisions (Burd & Hans, 2018; Thaman, 2002, 2011). This procedure generates what is known as a “reasoned verdict.”

Given the general complexity of the tasks jurors face, it is clear why some courts aim to assist jurors by structuring the decision making process or requiring reasons for jury verdicts, and why some believe that these changes might help to reduce bias and improve decision making. However, psychological theory suggests there is good reason to suspect that requiring jurors to produce reasons for their decisions after they have already rendered a verdict is not likely to promote stronger decision making or improve decision accuracy. Individuals are often unaware of and unable to report their cognitive processes, and when they are asked to do so, may not be able to report why they have made a decision, or how their attitudes have affected their decision (e.g., Nisbett & Wilson, 1977). When people do attempt to report on their cognitive processes, they are often inaccurate (Nisbett & Wilson, 1977). Ample research suggests that a person’s motivation may bias their reasoning (e.g., Kunda, 1990). Furthermore, research finds that when individuals are specifically asked whether they are relying on bias, they report that they are not (Salerno et al., 2021).

Although several countries require reasons for jurors’ verdicts (e.g., Csere, 2012; Thaman, 2011), little empirical research (Wiggins & Breckler, 1990; Sood, in press) has explored whether and how various verdict procedures differentially affect jurors’ judgments. In this article, we review several verdict procedures (i.e., general, special, and reasoned verdicts). We then discuss psychological literature that can inform these processes. Next, we provide the results of two experiments designed to examine which verdict procedure, if any, produces the strongest verdict decisions (i.e., most in line with the evidence). In addition, we tested whether certain reasoning requirements might mitigate the impact of racial/ethnic biases on juror decision making. We examined the impact of plaintiff race (Experiment 1) and ethnicity (Experiment 2) and verdict procedures on mock juror (Experiment 1, *N* = 267) and jury (Experiment 2, *N* = 292) decision making in a civil case of alleged defamation.

### 1.1. General and Special Verdicts in the U.S.

In the U.S., most jury trials utilize general verdicts. Here, jurors listen to the trial evidence, receive judicial instructions, and then retire as a group to deliberate on the evidence in private. Although juries are provided with judicial instructions on the law, the deliberation process itself is independent with few procedural guidelines. Generally, the only requirements are that the process must continue until a decision is made and often the decision must be unanimous. No reasons are required of the jury, and thus, little is usually known about the deliberation process or how jurors arrived at a decision.

In other circumstances, U.S. courts may use general verdicts with answers to written questions. In civil cases, juries may be tasked with answering a series of factual questions and then asked to render an ultimate judgment (i.e., liable vs. not liable) (Fed. R. Civ. P. 49(b)(1), n.d.). In criminal cases, too, questions might be posed to jurors when they are asked to assess a defense, or, in a case when a defendant has received multiple charges, the grounds on which the jury will convict (Nepveu, 2003). Further, questions are commonly used in capital jury trial sentencing to decide facts regarding aggravating or mitigating circumstances or to assess jurors’ perceptions of the defendant’s dangerousness (Nepveu, 2003).

A third category of verdicts used in U.S. civil trials is the special verdict, which shares some similarity to reasoned verdicts utilized abroad and to general verdicts with answers to written questions. With special verdicts, the jury answers several factual questions pertaining to the case. The jury does not decide the verdict; instead, the judge uses the responses to render the ultimate verdict (Fed. R. Civ. P. 49, n.d.). Special verdicts are meant to help jurors identify and organize key pieces of information during trial (Casper, 1993). In addition, special verdicts might help jurors by limiting the effect of confusing judicial instructions (Stephens, 1987). However, they have been subjected to sharp critique as well, as they are seen by some as “another means utilized by courts to weaken the constitutional power of juries and to vest judges with more power to decide cases according to their own judgments” (Ginsburg, 1965, p. 258, citing Justices Black and Douglas objecting to Fed. R. Civ. P. 49, n.d.). In this way, some argue that special verdicts disempower juries while further empowering judges. Importantly, special verdicts are not used in U.S. criminal cases, as leaving the ultimate decision to the judge appears to violate defendants’ Sixth Amendment right to have a jury of peers make the ultimate determination (Nepveu, 2003, citing *United States v. Gaudin*, 1995).

### 1.2. Reasoned Verdicts

One potential way the U.S. could adapt special verdicts to criminal court is through the use of reasoned verdicts. Under reasoned verdict procedures, juries still reach the ultimate verdict, as do juries deciding a general verdict, including a general verdict with answers to written questions. However, they also provide substantiated reasons in support of their verdict decisions (Burd & Hans, 2018; Thaman, 2002, 2011). Internationally, reasoned verdicts have been increasingly adapted both by classic juries composed entirely of lay citizens (e.g., Russia, Spain; Csere, 2012; Thaman, 1997, 2011) and by mixed courts, where professional and lay judges deliberate together (e.g., France, Italy: Cohen, 2016; Hans & Jolivet, 2016). The specific procedures vary. Spain has a classic jury system, but jurors do not deliberate freely before rendering a verdict. Instead, the judge provides jurors with a series of “yes” and “no” questions regarding the facts of the case, the charges, and possible defenses, along with a summary of the case from the judge (Thaman, 2011). Jurors are meant to deliberate on and take a vote for each of the questions posed to them (Thaman, 2011). For each question, they provide concise reasons indicating why they found each fact to be proved or not proved and identify which evidence they relied upon in making their decision (Ley Orgánica de 22 de Mayo, 1995; Thaman, 1997). The jury also provides a general vote of “guilty” or “not guilty” (Thaman, 2011). Jurors vote on each question, and verdicts are decided by majority rule (Jimeno-Bulnes, 2007, 2011). These reasoned verdict procedures are based on the assumption that such verdicts will promote strong jury decision making, increase transparency in the decision making process, and increase reviewability on appeal. However, little empirical research has informed this debate.

### 1.3. Examining Special, Reasoned, and General Verdicts Through a Psychological Lens

In practice, support for special verdicts is mixed. Proponents of special verdicts argue that adding questioning procedures could better focus deliberations by helping jurors to identify key facts within a case and discouraging jurors from taking a holistic approach to their decision making (Henderson et al., 1995). A holistic approach may encourage reliance on general impressions and biases, whereas the item-by-item approach required by special verdicts or general verdicts with answers to written questions may invoke greater reflection on the evidence. Further, by asking jurors to answer factual questions rather than delivering a general verdict, special verdicts might make it less likely that jurors tailor their responses to the questions based on their desired outcome of the case (Henderson et al., 1995). As Clermont (2018) argues, jury decisions are complicated and require applying a standard of proof to each element, so jurors should not merely answer an overall question. Special verdicts may help with the process, such as by organizing testimony and legal rules and by increasing juror comprehension of evidence (Casper, 1993). The reasoning requirement might compel jurors to think more carefully about their decisions (Jimeno-Bulnes, 2007). Further, some suggest that the reasoning requirement might encourage jurors to evaluate the evidence by comparing it to the prosecution’s and the defendant’s versions of events (e.g., a defendant claiming a stabbing was an accident versus the alternative possibility that the stabbing was intentional; Thaman, 1997). Interestingly, a study of actual jurors who received what researchers referred to as a special verdict form appreciated them and reported feeling as if they had reached a correct decision more than jurors who did not use such a form (Heuer & Penrod, 1994).

Moreover, Sood (2021) has identified potential negative consequences of general verdicts for criminal defendants, explaining that a jury’s general verdict might hide jurors’ biased factfinding and misunderstandings or misapplications of law. Sood (2023) gathered information about views of general verdicts and general verdicts with answers to written questions from more than 1600 respondents, including members of the public and legal stakeholders such as judges, prosecutors, defense attorneys, and civil litigators. Respondents expressed similar levels of support for general verdicts and general verdicts with answers to written questions in both criminal and civil trials. Interestingly, prosecutors (and not criminal defense attorneys) expressed the greatest opposition to general verdicts with answers to written questions in criminal trials. Further, judges of color were more supportive of general verdicts with answers to written questions in criminal trials than White judges. Reasons for supporting general verdicts with answers to written questions varied, with many respondents believing that they would help jurors understand and apply the law.

As for the effects of special verdicts in civil cases, most civil litigators (65% plaintiff attorneys and 59% defense attorneys) thought that it depended on the case, but those who expressed an opinion said that special verdicts generally favor the defense in civil cases (Sood, 2021). However, other scholars argue that special verdicts might, in fact, be biased in favor of the plaintiff (Lombardero, 1995) because they prevent the jury from rejecting multi-element claims wholesale.

Opponents of special verdicts argue that free deliberation is inherently filled with debate as jurors discuss key issues to come to a unanimous decision (Abramson, 2015). Further, some legal commentators argue that providing reasons is unnecessary; because general verdicts are based on applying judicial instructions to the evidence presented at trial, the reasons behind jury verdicts are already clear (Lempert, 2007, 2015). In fact, research suggests that deliberating juries tend to perform very well across a variety of metrics (Reed et al., 2024).

In one of the few previous studies of jury verdict format, a mock juror experiment examined the impact of special verdicts on civil juror decisions (Wiggins & Breckler, 1990). In that work, participants watched a trial video and gave either a general liability verdict or a special verdict in which they answered a set of factual questions. Then, participants completed measures of their impressions of the parties. Jurors made similar liability decisions regardless of the type of verdict (Wiggins & Breckler, 1990). However, there were differences in damage awards, with jurors awarding higher compensatory damages when using the special verdict form than general verdict form. Wiggins and Breckler also found that impressions of the parties and impressions of the parties’ cases influenced verdicts, such that as participants’ positive perceptions of a party and their case increased, they were more likely to issue a verdict in favor of that party. We build on this work by (1) examining several types of verdict procedures (Study 1); (2) examining if these effects differ depending on plaintiff race (Experiment 1) and ethnicity (Experiment 2); and (3) examining the impact of deliberation (Experiment 2).

In another set of studies, Sood (in press) varied the verdict format in mock criminal cases given to participants. Some were asked to give a general verdict, whereas others were guided to a verdict decision by a sequential set of questions. Sood labeled this condition the “special verdict” condition, but unlike true special verdicts in U.S. courts, the participants in this condition also reached a final verdict. In the first experiment, the special verdict condition did not help participants better understand the law, but nonetheless the defendant was acquitted more frequently when mock jurors made their judgments in the special as opposed to the general verdict condition. In a second experiment featuring a mercy killing, where juror emotions might tilt them toward acquittal, the defendant was more likely to be convicted of a lesser crime in the general as opposed to the special verdict condition—but only if he was a White or Black Christian and not if he was a Muslim. Based on these studies, special verdicts may predict greater leniency than general verdicts, but only for some defendants.

More general psychological research also gives reason to question the efficacy of special verdicts. We review such research now.

#### 1.3.1. The Story Model of Jury Decision Making

The story model of juror decision making is considered one of the most accurate accounts of how jurors make decisions when determining guilt using a general verdict (Levett & Devine, 2017; Vidmar & Hans, 2007). The story model suggests that as jurors listen to testimony and evidence at trial, they begin to form a narrative that they feel best describes what they are hearing (Pennington & Hastie, 1986, 1988, 1992, 1993). In the process of deliberation, the story model suggests that jurors discuss and analyze their narratives with one another, and then, through group discussions, create or decide on a narrative that is the best fit for the evidence (Pennington & Hastie, 1993). When deciding on guilt, they compare their narrative to the verdict category options and choose the verdict that most aligns with their conception of the trial narrative and best accounts for the case facts (Pennington & Hastie, 1992).

Research regarding the story model suggests that jurors might make decisions most in line with the evidence, and with more confidence, when allowed to structure the evidence according to a narrative and to deliberate freely (Pennington & Hastie, 1992). In one study, researchers found that mock jurors made stronger decisions (more in line with the evidence) when evidence was presented in chronological narrative form, rather than separated by legal issue (Pennington & Hastie, 1992). Generally, research regarding the story model suggests that, without prompting, mock jurors engage in deep deliberative reasoning, make inferences, use analogies, and compare alternatives (Pennington & Hastie, 1993).

In contrast, research suggests that when mock jurors are presented with evidence item by item in the context of a criminal trial, they are less likely to make judgments in line with the preponderance of the evidence compared to jurors who are presented with evidence in story order. Further, Pennington and Hastie (1992) found that mock jurors were less confident in their judgments when evidence was presented item by item rather than in story form. This item-by-item presentation somewhat mirrors how evidence may be presented to jurors when tasked with using reasoned or special verdict forms: Such verdict forms often separate questions of fact from one another (Fed. R. Civ. P. 49(b)(1), n.d.; Nepveu, 2003), and reasoned verdict forms in particular lack standardization (e.g., Thaman, 2011). It is possible that reasoned and special verdict forms might reduce decision quality by focusing jurors on specific questions rather than the more holistic narrative (Pennington & Hastie, 1992).

#### 1.3.2. Predecisional Distortion, Motivated Reasoning, and Coherence-Based Reasoning

Predecisional distortion occurs when individuals distort new incoming information to fit with a preexisting belief or preference (Russo et al., 1998). Research regarding predecisional distortion and motivated reasoning suggests that a reasoning requirement may not ensure stronger juror decisions compared to traditional general verdicts—especially if the reasoning is given after jurors reach a general verdict, as discussed below. That is, the reasons that jurors generate might be influenced by the same biases as their general verdicts. Further, research suggests that predecisional distortion is more likely to occur when the incoming information is ambiguous and could be interpreted several ways (Russo et al., 1996). In research exploring predecisional distortion in mock trial settings, Carlson and Russo (2001) found that mock jurors distorted incoming evidence to conform with their preexisting beliefs in both civil and criminal cases, even when warned not to do so and admonished in judicial instructions. The likelihood of engaging in predecisional distortion was even greater when jurors were more confident in a leading verdict preference (Carlson & Russo, 2001).

Like predecisional distortion, motivated cognition and reasoning can distort individuals’ perceptions of new evidence in light of their verdict preference (Kahan, 2013). In a legal context, Sood (2015) found that participants perceived illegally obtained evidence and even case law differently depending on what case outcome they preferred. Specifically, participants acting as judges were more likely to interpret illegally obtained evidence as permissible when the crime was more severe compared to less severe.

Research regarding predecisional distortion and motivated reasoning suggests that reasoning requirements may not be more successful in promoting verdict accuracy or reducing bias compared to general verdicts. If the reasons are required after jurors are already likely to have a leading preference for a verdict, a reasoning requirement may not actually reduce bias in jurors’ reasoning or promote decision accuracy. Instead, the reasoning requirement may increase juror confidence without increasing the deliberative processing of evidence before an intuitive decision regarding verdict is made.

Research regarding coherence-based reasoning (Simon, 2004) also suggests that the reasoning requirement may not protect jurors from biased decision making. According to Simon (2004), coherence-based reasoning is likely at play during complex decision making tasks. This model of reasoning proposes that decisions are made as a product of cognitive processes that occur bi-directionally: “Premises and facts both determine conclusions and are affected by them in return” (Simon, 2004, p. 511). Further, Simon (2004, p. 511) argues that “[a] natural result of this cognitive process is a skewing of the premises and facts toward inflated support for the chosen decision.” According to this model, as one option becomes more and more favored, it is perceived as increasingly strong while the alternative option is perceived as weaker and weaker, which increases confidence in the leading choice and makes it seem like an obvious option.

These theoretical assumptions have been empirically tested utilizing mock jury paradigms to examine coherence-based reasoning (Simon, 2004; Simon et al., 2015). Once jurors begin to favor one verdict over another, their interpretation of new evidence is likely to be distorted to conform to their preference, and the reasons they provide after making their decisions will likely be formed completely post hoc, all to align with their preferred verdict.

### 1.4. Timing of Reasoning

Although research might suggest that all reasoning requirements might be ineffective in improving verdict accuracy, the timing of the reasoning might further exacerbate the concerns described previously. Most countries that use reasoned verdicts tend to require the reasoning after the verdict is already reached, not before. As discussed previously, people are not always good at identifying their motivations post hoc. It is possible that changing the timing of the reasoning to require it before the decision is rendered might result in verdicts that are reflective of the reasoning, rather than reasoning that is reflective of the verdict. The limited research on this topic suggests timing matters, at least to the emotionality of the decision makers. Liu (2018) manipulated the timing of reasoning and assessed mock judges’ emotional responses. Requiring mock judges to provide reasons before rendering a judgment substantially reduced the effect of emotion compared to those who did not provide reasons before making a determination of guilt. On the other hand, providing reasons after rendering a judgment did not reduce the impact of emotional bias (e.g., Haidt, 2001; Liu, 2018). Therefore, timing of reasoning might be important.

### 1.5. Research Overview

Jury decision making using general verdicts has considerable strengths. Juror reasoning during deliberation is often complex, and mock jurors make connections and inferences based on trial evidence, properly reference the law in their discussions, and often are able to accurately resolve questions regarding case facts (Ellsworth, 1989). Jury research indicates that jurors generally make sound decisions. Civil juries perform relatively well against several metrics without interventions (Reed et al., 2024). Moreover, U.S. judges are often in agreement with juries’ criminal and civil verdicts, and the strength of the evidence presented at trial is the best predictor of juries’ verdicts (Eisenberg et al., 2005).

Yet, as we have described, jury decision making using general verdicts can also be susceptible to biases. Research shows that mock jurors have better recognition for evidence that aligns with their verdict preference, and mock jurors at times incorrectly recall information that was never presented if it fits their narrative of the trial evidence (Pennington & Hastie, 1988). Jurors may fail to discuss important topics, particularly if those topics relate to a verdict that no single juror is favoring (Ellsworth, 1989). Juror discussions can also be “verdict-driven” rather than “evidence-driven” (Hastie et al., 1983), and verdict-driven deliberations are more likely than evidence-driven deliberations to end in a hung jury (Hannaford et al., 1998). It is possible that requiring reasons could promote evidence-driven deliberations, although reasoning requirements are not necessarily free of bias. This overall picture of decision making strength yet susceptibility to bias suggests the value of exploring the effects of structured approaches to jury verdicts.

Despite the relative paucity of empirical research regarding reasoned verdicts, several countries have established a reasoning requirement for jurors. Given the research described above regarding how individuals and groups generally make decisions, it is important to investigate how jurors make decisions across these varied contexts and verdict procedures. Across two mock civil jury experiments, we tested the influence of verdict procedure with and without reasoning requirements and examined whether such verdict procedures differentially impact mock jurors’ decisions.

## 2. Experiment 1

In Experiment 1, we tested four verdict procedures (general verdict vs. special verdict vs. reasons before a general verdict vs. reasons after a general verdict) to examine whether mock jurors reached similar verdicts across these different verdict procedures. This method allowed us to test two versions of reasoned verdicts that varied in timing—before the general verdict (“reasons before”) or after the general verdict (“reasons after”). Further, to test whether these verdict procedures might improve juror decision accuracy by reducing bias, we manipulated plaintiff race (White vs. Black). Our predictions focus on liability verdicts and damage award determinations.

**Hypothesis** **1.** *American juries tend to exhibit bias against Black individuals in the courtroom. For example, researchers examined over 9000 civil jury trials in Illinois and found that Black litigants (plaintiffs and defendants) lost more often than White litigants, and Black plaintiffs were awarded smaller sums (Chin & Peterson, 1985), mirroring recent research that finds civil jurors award fewer damages to Black compared to White plaintiffs (Cardi et al., 2020; Girvan & Marek, 2016). Therefore, we expected jurors to demonstrate bias against Black plaintiffs, such that jurors would render more liability verdicts for White than Black plaintiffs and give higher damage awards.*

**Hypothesis** **2.** *We also predicted a main effect of verdict procedure on mock jurors’ decisions on damages. Drawing on Heuer and Penrod (1994) and especially Wiggins and Breckler (1990), we did not expect differences in liability verdicts based on verdict procedure. However, we hypothesized that damage awards would be higher in the special verdict condition than in the general verdict condition (Wiggins & Breckler, 1990).*

**Hypothesis** **3.** *We also expected an interaction between plaintiff race and verdict procedure. Because research suggests that providing reasoning before a verdict reduces the emotional response (Liu, 2018), we expected that providing reasons before rendering a verdict would result in reduced racial bias. Specifically, we expected that all participants would show bias against a Black plaintiff (i.e., finding for the defendant) except in the reasons before condition. As no known prior research has examined the potential for true special verdicts to reduce racial bias, no specific predictions were made regarding plaintiff race and the special verdict procedure.*

**Hypothesis** **4.** *It was also expected that participants’ overall impressions of the plaintiff and defense cases and their impressions of the plaintiff and defendant would predict jurors’ judgments, such that the more favorable one’s impressions of the plaintiff, the more likely they would be to find for the plaintiff (Phalen et al., 2025). Importantly, it was hypothesized that, across all conditions except for those in the reasons before conditions, jurors’ experience of emotions would independently predict their finding for the plaintiff or defendant. Experiment 1 was approved by the institutional ethics committees of Cornell University.*

### 2.1. Method

#### 2.1.1. Participants

Four hundred twelve individuals were recruited for the current research. Two hundred thirty-five (57%) were recruited via snowball and convenience sampling by email, social media, and word of mouth. These participants volunteered their time and were not compensated for their participation. One hundred seventy-seven participants (43%) were recruited utilizing Mechanical Turk (Paolacci et al., 2010) via Turk Prime (Litman et al., 2017). Individuals in this sample who completed the full survey (*n* = 140) were compensated USD2.00 for their participation. Data from across the various samples were collected concurrently. We excluded participants if they failed more than one of the three attention check questions (*n*_Convenience_ = 75; *n*_MTurk_ = 70), resulting in a final sample of 267 participants (*n*_Convenience_ = 160; *n*_MTurk_ = 107). We did not exclude participants based on the race manipulation check failure as many participants failed the manipulation check (*n* = 118, 44.19%). Manipulation check failure did not differ based on condition: *χ*^2^ = 1.81, *p* = 0.18 (*n*_White Plaintiff_ = 66, 48.20%; *n*_Black Plaintiff_ = 52, 40.00%). We discuss the implications of this decision in the Study 1 Discussion. Although the two samples differed demographically (see Table 1), there were no differences in verdict by sample (*p* = 0.99). Further, evidence suggests college-aged and non-college-aged samples make mock legal decisions similarly (e.g., Nuñez et al., 2011). Therefore, we collapsed all analyses across the samples.

#### 2.1.2. Design

This study employed a 4 (Verdict procedure: general verdict vs. special verdict vs. reasons before vs. reasons after) × 2 (Plaintiff race: White plaintiff vs. Black plaintiff) fully crossed between-subjects design.

#### 2.1.3. Materials^1^

*Case Summary*. The case, based on a fact pattern used by Wiggins and Breckler (1990), described a female plaintiff suing a male defendant for defamation. The plaintiff had worked as a maid for the defendant for two years until the defendant implied that the plaintiff stole an expensive piece of jewelry. The missing jewelry was later found in the defendant’s home. The plaintiff testified she applied for many new jobs but was unemployable due to a negative character reference from the defendant. Specifically, she claimed that the defendant defamed her to a potential employer (the general manager of a country club) when he detailed allegations of theft and reported perceiving her behaviors as suspicious. After the evidence, participants read judicial instructions detailing the claims against the defendant, the standard of proof, and possible defenses.

*Plaintiff Race.* The plaintiff’s race was manipulated by changing the plaintiff’s name in the materials. In the Black plaintiff condition, the plaintiff was named Latoya Jackson, whereas in the White plaintiff condition, the plaintiff was named Jennifer Becker.

First names were selected from a database of 4250 first names (Tzioumis, 2018). To choose a typical first name for both African and European Americans, the data was first sorted by percentage of individuals holding these first names within each racial category from most to least and then compared these names to overall number of occurrences of the name across the general population. Latoya was chosen as 91.18 percent of individuals with that name were African American, and 93 individuals in the data set were named Latoya. Using the same process, Jennifer was chosen; 19,356 women in the database were named Jennifer, and of those, 94.44% were European American. Next, the name Jackson was chosen as the last name for the African American plaintiff, based on a frequency table by Comenetz (2016) based on the 2010 Census Data (U.S. Census Bureau, 2010). Jackson was chosen as it ranked 19 out of all African American surnames, with 708,099 individuals having Jackson as a last name (Comenetz, 2016). Becker was chosen as the last name of the European American plaintiff as it ranked 315 of surnames, and 96.4% of individuals with this last name were classified as White (Gaddis, 2017, citing U.S. Census Bureau, 2010).

*Verdict Procedure*. Verdict procedure was manipulated through a verdict form. For the general verdict, participants were simply asked for a liability determination and, if they found the defendant liable for defamation, a damage award. For the special verdict, jurors answered four yes/no questions about the findings, but did not render an ultimate judgment of liability (i.e., *Did the Plaintiff prove by a preponderance of the evidence that the Defendant made a defamatory statement against her?* [Yes: *n* = 48, 67.61%]; *Did the Plaintiff prove by a preponderance of the evidence that the defamatory statement injured her?* [Yes: *n* = 48, 67.61%]; *Did the Plaintiff prove by a preponderance of the evidence that the Defendant made the defamatory statement with malice toward the Plaintiff, or with a reckless disregard for her interests?* [Yes: *n* = 35, 49.30%]; and *Did the defendant prove by a preponderance of the evidence that the defamatory statement was true?* [No: *n* = 59, 83.10%] Table 2). In both reason conditions, participants completed the general verdict plus answered two open-ended questions about the legal and factual support for either finding (one pro-plaintiff, one pro-defendant). The timing of this reasoning occurred either before (reasons before) or after (reasons after) they decided on a general verdict.

#### 2.1.4. Measures

*Attention and Manipulation checks*. Mock jurors were asked three multiple-choice attention check questions: *What crime was the defendant accused of?*; *Where was the missing jewelry found?*; and *How long did Latoya Jackson/Jennifer Becker work as a maid for the Morgan family?* Mock jurors were also asked a race manipulation check: *What is the race/ethnicity of the Plaintiff, Latoya Jackson/Jennifer Becker, who worked as a maid for the Morgan family?*

*Affect*. Mock jurors completed an affect questionnaire containing several items from the Positive and Negative Affect Schedule (PANAS; Watson et al., 1988). Participants were asked to indicate how they felt, right now, using a scale ranging from 1 (*Very slightly to not at all*) to 5 (*Extremely*) for a large variety of emotions (e.g., anger, anxiety, calm, disgust, surprised, upset).

*Verdict and verdict confidence*. Mock jurors each rendered a verdict using their assigned verdict procedure (i.e., general verdict vs. special verdict vs. reasons before vs. reasons after). Mock jurors were asked to rate their confidence in their decision from 1 (*Not at all confident*) to 7 (*Very confident*).

*Damage awards*. Participants who found for the plaintiff were asked to choose an appropriate damage award, to rate their confidence in the assigned award amount from 1 (*Not at all confident*) to 7 (*Very confident*), and to indicate how difficult it was to pick an exact award amount from 1 (*Not at all difficult*) to 7 (*Extremely difficult*). Special verdict forms provided guidance to mock jurors regarding whether to assess damages based on their responses to the series of fact-based questions. Mock jurors who responded “yes” to the first three questions and “no” to the fourth were instructed to award damages (Table 2).

*Impressions of the plaintiff, defendant, and their cases*. Mock jurors were asked to indicate their impressions of the plaintiff and defendant using seven-point bipolar scales for characteristics such as *Immoral*–*Moral* and *Unlikeable*–*Likeable*. Mock jurors indicated their impressions of the plaintiff’s and defendant’s cases using a seven-point bipolar scale for items such as *Unpersuasive*–*Persuasive* and *Unbelievable*–*Believable*.

*Meta-cognitive reflection.* Mock jurors also responded to general questions pertaining to their participation, including: *How motivated were you while reading this trial summary?* and *How motivated were you while determining an award for the Plaintiff, Latoya Jackson’s/Jennifer Becker’s, suffering?* on a scale from 1 (*very little)* to 7 (*highly*); *How much cognitive effort did you expend while reading this trial summary?* and *How much cognitive effort did you expend while determining an award for the Plaintiff, Latoya Jackson’s/Jennifer Becker’s, suffering?* on a scale from 1 (*very little)* to 7 (*high*); *How much of a role did punishment of the Defendant, John Morgan, factor into your award decision?* and *How much of a role did economic losses of the Plaintiff, Latoya Jackson/Jennifer Becker, factor into your award decision?* on a scale from 1 (*none)* to 7 (*a great deal*). Only jurors who found for the plaintiff answered questions pertaining to an award.

*Cognitive Reflection Test (CRT)*. Participants completed the CRT (Frederick, 2005), which assesses individual’s ability to curb fast, intuitive, wrong responses to questions, and to instead answer using more deliberative processing to respond correctly.

*Demographic questionnaire*. Mock jurors then answered several demographic questions pertaining to citizenship, age, sex, ethnicity, education, number of STEM classes taken, and political orientation.

#### 2.1.5. Procedures

All participation occurred online using the Qualtrics platform. After providing informed consent, participants were randomly assigned to condition. Mock jurors read the brief case summary and judicial instructions. They then completed the affect questionnaire. Next, participants rendered a verdict using the randomly assigned procedure. Mock jurors who found for the plaintiff were asked to award damages and answered several questions pertaining to the damages awarded, including confidence in their award decision, and the difficulty of deciding on an exact award.

Next, mock jurors answered questions regarding their overall impressions of the plaintiff and the plaintiff’s case, and perceptions of her suffering; overall impressions of the defendant and the defendant’s case, and the extent they believed the defendant caused the plaintiff suffering; how motivated they were while reading the trial and when determining an award (if applicable); how much cognitive effort they expended reading the trial and in determining an award (if applicable); if applicable, how much punishing the defendant played a role in their award, and how much the plaintiff’s economic losses factored into their award; the Cognitive Reflection Task; and basic demographic questions.

### 2.2. Analytic Plan

All analyses were conducted in R and RStudio (v. 2023.03.0+386; R Core Team, 2023). Analyses were conducted using the stat package, unless otherwise noted.

#### 2.2.1. Factor Scores

Confirmatory factor analyses were conducted for items of positive and negative affect (Table A1), perceptions of the defendant and his case, and perceptions of the plaintiff and her case (Table A2). Principal axis factor analyses were conducted for each of these categories, and a single factor was extracted for each category. These standardized scores were then used for subsequent analyses. We conducted these analyses in R using the lavaan package (Rosseel, 2012).

Next, using these composite scores, difference scores were created for perceptions of the two sides’ cases (perceptions of the plaintiff—perceptions of the defendant) and perceptions of the plaintiff’s and defendant’s cases (perceptions of the plaintiff’s case—perceptions of the defendant’s case). These difference scores were used in subsequent analyses.

#### 2.2.2. Verdict and Verdict Confidence

We conducted a series of logistic regressions to test for a three-way interaction between the independent variables (plaintiff race and verdict procedure) and sample and to investigate whether plaintiff race and/or verdict procedure independently predicted verdict, over and above participants’ overall impressions of the plaintiff and defendant and positive and negative affect. We also examined whether the perceptions of the plaintiff’s case and the defendant’s case, the perceptions of the plaintiff and defendant, and positive and negative affect predicted verdict. We then conducted a series of conceptually similar linear regressions with verdict confidence as the dependent variable.

#### 2.2.3. Damage Awards

Participants were only asked to provide damages if they found for the plaintiff (*n* = 176, 65.91%). Therefore, for damage award analyses, we examined the damage awards made by plaintiff jurors. Across all conditions, damages ranged from USD 0 to USD 1,000,000 (*M* = USD 72,382.43, *SD* = USD 154,598.83, *Median* = USD 30,000, *IQR* = USD 54,000). Only two people awarded zero dollars in damages. As is typical with damage awards (Greene et al., 2001; Phalen et al., 2021), damage awards were positively skewed. To account for that skew, we conducted regressions assuming a gamma distribution (Salerno et al., 2021). We added a constant of one to each value to account for zeros.

### 2.3. Results

#### 2.3.1. Verdict and Verdict Confidence

Across all conditions, 66.2% of participants found for the plaintiff. These rates parallel other similar research (Wiggins & Breckler, 1990). A logistic regression model that included the three-way interaction between the independent variables (plaintiff race and verdict procedure) and sample did not perform significantly better than a model that did not include sample, *χ*^2^ = 8.18, *p* = 0.42. Further, the overall three-way interaction was not significant, Wald = 0.62, *p* = 0.89. All subsequent analyses were performed on the total sample.

**Figure 1:**
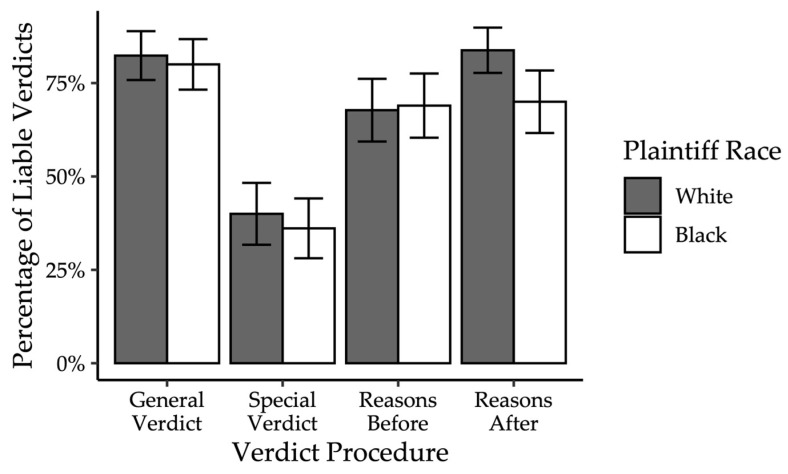
Percentage of Mock Jurors Who Found for the Plaintiff Across Conditions. *Note.* Error bars represent standard errors.

As discussed above, a logistic regression analysis was conducted to test for a three-way interaction between the independent variables (plaintiff race and verdict procedure) and sample. Analyses revealed that the overall model was significant, *χ*^2^ = 134.56, *p* < 0.001 (Figure 1).

Next, a logistic regression analysis was performed to investigate whether plaintiff race and/or verdict procedure independently predicted verdict, over and above participants’ overall impressions of the plaintiff and defendant and positive and negative affect. Analyses revealed that the overall model was significant, *χ*^2^ = 126.39, *p* < 0.001. Plaintiff race did not significantly predict verdict, *b* = −0.08, *SE* = 0.39, Wald = 0.16, *p* = 0.83, odds ratio = 0.92. An exploratory model identical to the model discussed prior, but with the inclusion of participants’ CRT scores, produced similar results, and the CRT was not predictive of verdict, *b* = −0.24, *SE* = 0.18, Wald = 1.76, *p* = 0.18, odds ratio = 0.79.

However, verdict procedure significantly impacted verdict, Wald = 26.27, *p* < 0.001. Participants in the special verdict conditions were significantly less likely to find for the plaintiff compared to participants in all other verdict procedure conditions. Participants who rendered a general verdict *(b* = 2.56, *SE* = 0.57, Wald = 21.75, *p* < 0.001, *OR* = 12.94), provided reasons before a general verdict *(b* = 1.57, *SE* = 0.53, Wald = 21.75, *p* = 0.003, *OR* = 4.78), or provided reasons after rendering a general verdict *(b* = 2.48, *SE* = 0.56, Wald = 20.90, *p* < 0.001, *OR* = 11.94) were significantly more likely to find for the plaintiff compared to those in the special verdict conditions. There was no significant interaction between plaintiff race and verdict procedure, Wald = 2.70, *p* = 0.44. The verdict procedure finding stands in contrast to Wiggins and Breckler (1990) who found that jurors were equally likely to find for the plaintiff when rendering either a special or general verdict.

Consistent with Wiggins and Breckler (1990), mock jurors’ perceptions of the plaintiff’s and defendant’s cases independently predicted verdict, *b* = 1.61, *SE* = 0.33, Wald = 24.08, *p* < 0.001, odds ratio = 4.99. However, in contrast with Wiggins and Breckler (1990), participants’ perceptions of the plaintiff and defendant did not significantly predict verdict, and neither did their positive and negative affect, *ps* > 0.14.

Given that we did not exclude participants based on manipulation check failure, we replicated this logistic regression analysis with whether participants failed the manipulation check included in the model. Including manipulation check failure in the model did not significantly improve the model performance, *χ*^2^ = 0.77, *p* = 0.68. The results discussed above replicate when performance on the manipulation check is included in the model.

We also examined verdict confidence. Contrary to predictions, verdict confidence was not significantly influenced by verdict procedure (*F*(3, 226) = 0.78, *p* = 0.51, *np*^2^ = 0.01), plaintiff race (*F*(1, 226) = 0.11, *p* = 0.75, *np*^2^ = 0.00), or their interaction (verdict confidence: *F*(3, 226) = 0.08, *p* = 0.99, *np*^2^ = 0.00).

#### 2.3.2. Damages

**Figure 2:**
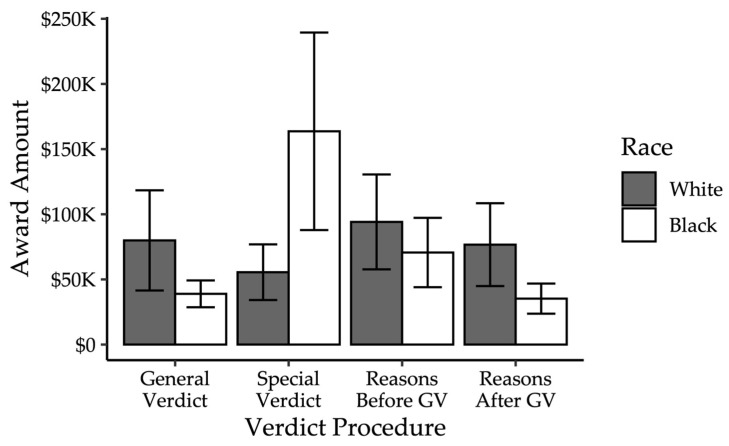
Average Damages Awarded Across Conditions. *Note.* Error bars represent standard errors.

Contrary to expectations, there was also no effect of the manipulations on damage awards. The overall model was not significant, χ^2^ = 31.98, *p* = 0.27. Neither verdict procedure (χ^2^ = 3.71, *p* = 0.29), plaintiff race (χ^2^ = 1.51, *p* = 0.21), or their interaction (χ^2^ = 4.71, *p* = 0.31) significantly predicted damages, as shown in Figure 2.

#### 2.3.3. Exploratory Analyses

Exploratory analyses were conducted to investigate the impact of plaintiff race and verdict procedure on participants’ motivation and cognitive effort expended while reading the case summary. A multivariate analysis of variance revealed no significant differences for race, Pillai’s trace = 0.01, *F* = 0.64, *df* = (2,226), *p* = 0.53, *ηp*^2^ = 0.006, verdict procedure: Pillai’s trace = 0.02, *F* = 0.74, *df* = (6,454), *p* = 0.62, *ηp*^2^ = 0.01, or the interaction, Pillai’s trace = 0.01, *F* = 0.56, *df* = (6,454), *p* = 0.76, *ηp*^2^ = 0.007.

Exploratory analyses were also conducted to investigate the impact of plaintiff race and verdict procedure on perceptions of the parties. There were no significant differences for race, *F*(1, 231) = 1.91, *p* = 0.17, *ηp*^2^ = 0.008, verdict procedure: *F*(3, 231) = 1.22, *p* = 0.30, *ηp*^2^ = 0.02, or the interaction, *F*(3, 231) = 0.10, *p* = 0.96, *ηp*^2^ = 0.001. Finally, exploratory analyses were conducted to investigate the impact of plaintiff race and verdict procedure on perceptions of each party’s case. There were no significant differences for race, *F*(1, 231) = 0.17, *p* = 0.68, *ηp*^2^ = 0.001, verdict procedure: *F*(3, 231) = 0.91, *p* = 0.44, *ηp*^2^ = 0.01, or the interaction, *F*(3, 231) = 0.12, *p* = 0.95, *ηp*^2^ = 0.001.

### 2.4. Discussion

Given the contemporary movement towards a reasoning requirement for juries abroad, it is important to empirically investigate the impact of verdict procedures on jury decision making. The current study provides some evidence to suggest that general verdicts with reason-giving may not promote stronger decision making, that is, in line with the evidence, compared to general verdicts without reason-giving.

No evidence was found in support of Hypothesis 1 regarding plaintiff race. Specifically, plaintiff race did not influence verdict, verdict confidence, or damages. In the current study, plaintiff race was manipulated solely through differing names for the plaintiffs (i.e., not through photo manipulation). It is possible that mock jurors may not have noticed the plaintiff’s race. Even if they noticed the plaintiff’s race, it is possible that they responded in a socially desirable way in order to avoid presenting themselves as racist (Hunt, 2015; Salerno et al., 2023).

Though it was hypothesized that participants who rendered either a general or special verdict would decide the case in favor of the plaintiff at similar rates (Hypothesis 2), the special verdict procedure significantly reduced the likelihood of finding for the plaintiff. Importantly, mock jurors’ decisions were comparable when utilizing general verdicts that required them to provide reasons before or after rendering a general verdict. In the current study, the reasoning requirement did not appear to change jurors’ verdicts.

Reason-giving has been found to promote unbiased decision making in prior research when negative feelings towards a defendant are induced (Liu, 2018). Several key methodological and sample differences may explain the differential findings from the current study in comparison to Liu (2018). First, Liu (2018) sampled judges who differ extensively in training and experience compared to the lay individuals in the current study. Second, Liu (2018) exposed judges to potentially more affect-inducing offenses (corruption, theft), which may have elicited more affect compared to the alleged claim of defamation in the current study. Given that neither plaintiff race nor verdict procedure influenced mock juror affect, and that affect was quite low across all measures of emotions and conditions (e.g., *M_fear_* = 1.30, *SD* = 0.99, *Range* = 1–7), it follows that the verdict procedure manipulation was not predictive of affect, as affect was not induced by the mock trial stimuli.

Contrary to Hypothesis 3, there was no interactive effect of the plaintiff race manipulation and verdict procedure on mock jurors’ decisions. The lack of significant interaction might be attributed to the same reasons that the main effect of race was not significant—either the manipulation was unsuccessful, or participants were acting in socially desirable ways. However, it is still important to note that those in the special verdict conditions decided the case significantly differently compared to participants in all other conditions. The case summary presented was rather balanced, and even so, the majority of participants found for the plaintiff when rendering a general verdict, with or without reasons. In contrast, those responding to the questions in the special verdict procedure were substantially more likely to find for the defendant compared to those rendering a general verdict.

In line with Hypothesis 4 and similar research, perceptions of the defendant’s and plaintiff’s cases also significantly predicted verdict, independent of verdict procedure (Wiggins & Breckler, 1990)—although perceptions of the plaintiff and defendant themselves did not. This again suggests that jurors decided the case similarly across conditions with the exception of the special verdict conditions. Thus, in the current study, verdict procedures did not appear to affect jurors’ perceptions of the defendant’s or plaintiff’s cases.

Contrary to predictions and other research, verdict confidence did not vary across conditions (Heuer & Penrod, 1994; Wiggins & Breckler, 1990). Mock jurors were relatively confident, regardless of verdict procedure, though across all conditions, mock jurors were split regarding liability (i.e., approximately 66% found for the plaintiff). In addition, mock juror affect did not independently predict verdict. While the current case portrayed a sympathetic plaintiff, the case and claims presented did not seem to elicit strong moral emotions in mock jurors.

Lastly, mock jurors assessed damages similarly across conditions. While there was a wide range of damages awarded ranging from zero to one million, mock jurors generally assessed similar amounts, regardless of plaintiff race or verdict procedure.

The current study investigated mock juror, but not jury, decision making. It is important to explore the relative impact of the reasoning requirement on mock jurors and jurors who deliberate as a group. Further, the plaintiff race manipulation utilized in the current study did not produce differences attributable to bias, so we could not test whether the reasoning requirement could reduce bias in jurors or arbitrary decision making.

## 3. Experiment 2

Experiment 2 was designed to investigate the potentially interactive effects of verdict procedure (general vs. special verdict with reasoning) and plaintiff ethnicity (Latinx plaintiff vs. non-Latinx plaintiff) on mock jury decision making. The special verdict with reason-giving is akin to reasoned verdicts used abroad, including those involving criminal matters (e.g., Jimeno-Bulnes, 2021). Experiment 2 was designed to replicate and extend Experiment 1 in several ways.

First, we eliminated the timing manipulation in Experiment 2. In Experiment 1, there were no significant effects of reason-giving timing on mock juror decisions, plausibly because affect was not highly activated by the claim of defamation. Further, the timing of reason-giving was dropped in Experiment 2 to improve ecological validity: courts abroad that utilize reasoned verdicts require jurors to provide their reasoning after voting on individual issues within the trial (e.g., Spain; Thaman, 2011).

Second, we changed the plaintiff manipulation to focus on plaintiff ethnicity (Latinx vs. non-Latinx) in Experiment 2 instead of race (Black vs. White). Recent research finds that Latinx individuals are currently experiencing significant dehumanization (e.g., Pinto, 2019), and that laypeople may be more biased against Latinx individuals given recent media highlighting contentious debates regarding immigration (Armenta et al., 2022). We also strengthened the plaintiff ethnicity manipulation by adding a photo of the plaintiff instead of just manipulating the plaintiff’s name, and we added an ethnicity manipulation check question.

Lastly, to improve upon the limitations regarding ecological validity (e.g., Nuñez et al., 2011) in Experiment 1, Experiment 2 utilized a more immersive mock trial paradigm, including mock jury deliberation, and a sample of community members. Research suggests that although “the distribution of individual jurors’ pre-deliberation verdict preferences is a strong predictor of the jury’s final verdict,” the deliberation process sometimes has a significant impact on the final outcome of a jury trial (Salerno & Diamond, 2010). Further, the diversity of the jury can affect patterns in juries’ final verdicts (e.g., Devine et al., 2001).

We made several predictions regarding the influence of plaintiff ethnicity and verdict procedure on juror and jury judgments:

**Hypothesis** **1.** *We predicted that jurors would be more likely to render a liability judgment and give higher damage awards to the non-Latinx plaintiff than the Latinx plaintiff. We expected this pattern to occur in the individual juror decisions pre-deliberation and in the group deliberation decisions.*

**Hypothesis** **2.** *In line with findings from Experiment 1 regarding special verdicts, we predicted that juries’ verdicts would favor the defendant when using a special verdict with reason-giving compared to using a general verdict. Given that the modified special verdict with reason-giving also removes the jury’s ability to render an ultimate judgment, as was the case for the special verdict procedure utilized in Experiment 1, we predicted that those using the special verdict with reason-giving in Experiment 2 would be more likely to favor the defendant, even when reasons are provided.*

**Hypothesis** **3.** *Based on our findings from Experiment 1 regarding juror perceptions of the plaintiff and defendant, we predicted that more favorable perceptions of the plaintiff and her case would be associated with verdicts finding for the plaintiff, and in contrast, that favorable perceptions of the defendant and his case would be associated with verdicts finding for the defendant.*

### 3.1. Method

#### 3.1.1. Participants

We recruited 300 jurors across 58 juries. One hundred sixteen (53%) were recruited from the University of Wyoming SONA system and given 2 SONA credits for their participation. One hundred four (47%) were recruited utilizing Craigslist and were compensated USD 40 for their participation. All data were collected concurrently.

We applied exclusion criteria at the jury and juror level. Specifically, we excluded full mock juries if there was (a) randomization failure, *n_juror_* = 3 (1.00%), within *n_jury_* = 1 (1.72%); (b) if multiple jurors engaged in disruptive behavior (e.g., multiple sleeping jurors, refusing to deliberate), *n_juror_* = 10 (3.33%), within *n_jury_* = 2 (3.45%); or (c) the jury did not fill out the jury survey, *n_juror_* = 25 (8.33%), within *n_jury_* = 5 (8.62%). We excluded individual mock jurors if they (a) were clearly not paying attention (e.g., sleeping during deliberation), *n* = 1 (0.33%); (b) did not complete the post-deliberation survey, *n* = 15 (5.00%); (c) failed the manipulation check, *n* = 37 (12.33%; *n _White Plaintiff_* = 26; *n _Latinx Plaintiff_* = 11); or (d) self-reported that they were not a U.S. citizen (making them ineligible for jury duty), *n* = 3 (1.00%). Thus, our final sample size was 220 jurors (across 49 juries).

Unsurprisingly, participants across the subsamples were dissimilar in age, gender, education, and race/ethnicity (Table 3). However, as with Experiment 1, analyses revealed that participants across both subsamples decided on the verdict similarly, *p* = 0.09. This is in line with evidence that suggests that college-aged and non-college-aged samples make mock legal decisions similarly (e.g., Nuñez et al., 2011). Thus, the samples were collapsed, and all subsequent analyses were performed on one sample.

#### 3.1.2. Design

The currently study employed a 2 (Verdict procedure: general verdict vs. special verdict with reason-giving) × 2 (Plaintiff race: White vs. Latinx) fully crossed design.

#### 3.1.3. Materials^2^

*Case Summary*. Participants began by listening to an audio recording of a mock trial while viewing photos of each legal actor. The mock trial was based on the same case as Experiment 1 (Wiggins & Breckler, 1990) but was extended in several ways. The mock trial included opening and closing judicial instructions, opening and closing statements for the plaintiff’s and defendant’s attorneys, a character witness for the plaintiff, plaintiff testimony, two witnesses for the defense, and defendant testimony. The mock trial was approximately one hour and fifteen minutes long.

*Plaintiff Ethnicity.* Plaintiff ethnicity was manipulated via the plaintiff’s name and photograph. In the Latinx conditions, the plaintiff was named Ana Velasquez, whereas in the non-Latinx plaintiff conditions, the plaintiff was named Jennifer Becker. In addition, mock juries were presented with a picture alongside the plaintiff’s testimony (Ma et al., 2015). Stimulus sampling procedures were utilized for plaintiff photos (Wells & Windschitl, 1999).

*Verdict Procedure.* In the general verdict conditions, participants were simply asked to find for the plaintiff or the defendant. In the special verdict with reason-giving conditions, mock juries were asked to answer the same four yes-or-no fact-based questions from Experiment 1 (i.e., *Did the Plaintiff prove by a preponderance of the evidence that the Defendant made a defamatory statement against her?* [Yes: *n_juror_* = 45, 63.41%; *n_jury_* = 10, 37.04%]; *Did the Plaintiff prove by a preponderance of the evidence that the defamatory statement injured her?* [Yes: *n_juror_* = 19, 36.54%; *n_jury_* = 4, 33.33%]; *Did the Plaintiff prove by a preponderance of the evidence that the Defendant made the defamatory statement with malice toward the Plaintiff, or with a reckless disregard for her interests?* [Yes: *n_juror_* = 20, 38.46%; *n_jury_* = 4, 33.33%]; and *Did the defendant prove by a preponderance of the evidence that the defamatory statement was true?* [No: *n_juror_* = 19, 30.65%; *n_jury_* = 4, 28.57%]). Participants were instructed to only answer the last three questions if they answered yes to the first question. Two juries (*n_juror_* = 10) answered only the first and fourth questions. After answering each fact-based question, juries indicated how many jurors agreed with the majority decision, supplied the pieces of evidence on which they relied, and provided a succinct explanation of the reasons why they found each fact-based question proved or unproved.

#### 3.1.4. Measures

Mock jurors completed both pre- and post-deliberation questionnaires as individuals.

*Affect*. Mock jurors completed an affect questionnaire containing several items from the Positive and Negative Affect Schedule (PANAS; Watson et al., 1988), identical to Experiment 1, as both a pre- and post-deliberation measure.

*Verdict and verdict confidence*. In the pre-deliberation questionnaire, mock jurors were asked which verdict they favored and selected from among three options: “*Verdict for the plaintiff,*” “*Unsure,” or “Verdict for the defendant.*” As in Experiment 1, mock jurors were also asked to rate their confidence in their decision on a scale ranging from 1 (*not at all confident*) to 7 (*very confident*). After deliberation, juries also rendered a verdict (“*Verdict for the plaintiff,” “Verdict for the defendant,” or “Hung”*). In the case of a hung jury, the jury also provided the number of jurors who found for the plaintiff and defendant. Due to time constraints within the study, juries were considered hung if they could not reach a unanimous verdict within 30 min. This is in alignment with similar prior experimental methods utilizing mock jury deliberation (e.g., Koehler et al., 2016).

*Damage awards*. In the pre-deliberation questionnaire, mock jurors were asked how much money they would award the plaintiff, and to compare the amount of their award to a range of amounts ranging from “*Nil (basically nothing)*” to “*High amount of money.*” During deliberation, mock juries who found for the plaintiff were asked to determine compensatory and punitive damages. As in Experiment 1, special verdict forms provided guidance to mock jurors regarding whether to assess damages based on their responses to the series of fact-based questions. Mock jurors who responded “yes” to the first three questions and “no” to the fourth were instructed to award damages. In the post-deliberation questionnaire, mock jurors were asked several questions regarding their perceptions of the awards, if made. Questions included, for example, “*How much did you agree with the group’s award amount?*”

*Perceptions of deliberation*. In the post-deliberation questionnaire, mock jurors responded to several measures of their perceptions of the deliberation process. Questions included, for example, “*How much did you agree with the group verdict?*” on a scale from 1 (*not at* all) to 7 (*completely*) and “*How much power did you have during the deliberation*?” on a scale from 1 (*none*) to 7 (*a great deal*).

*Impressions of the plaintiff, defendant, and their cases*. Mock jurors were asked to indicate their impressions of the plaintiff and defendant using the identical measures as used in Experiment 1, as a post-deliberation measure.

*Juror comprehension checks*. To assess mock juror comprehension of the legal issues at trial, and the burden of proving each issue, mock jurors completed eight questions (e.g., “*The defendant made a defamatory statement.*”) and were asked to indicate which party had the burden of proving the issue (i.e., the plaintiff, the defendant, neither party), as a post-deliberation measure.

*Perceptions of lawsuits.* Mock jurors responded to several questions assessing their general perceptions of lawsuits (e.g., “How much worse is it to award too much money to an injured party than it is to award too little money?”) on a scale from 1 (*strongly disagree*) to 7 (*strongly agree*), post-deliberation (Meinhold & Neubauer, 2001).

*Manipulation check.* Mock jurors were asked to indicate the race/ethnicity of the plaintiff, and could choose from among several options (e.g., Black, Asian American, White, Latinx), post-deliberation. Participants were more likely to pass the manipulation check when the plaintiff was Latinx (*n* = 104, 92.90%), relative to White (*n* = 116, 82.30%), *b* = 0.11, *p* = 0.01. Ultimately, we excluded participants who failed the manipulation check.

*Demographic questionnaire*. Mock jurors completed a demographic questionnaire identical to that used in Experiment 1 post-deliberation.

#### 3.1.5. Procedures

Experiment 2 was conducted entirely online. Participants were recruited from Craigslist and via a university participant pool. Participants joined the study via Zoom. During each session, participants first provided consent, then listened to the mock trial detailed above. Next, they completed the pre-deliberation questionnaire, after which they formed a jury and participated in deliberation in accordance with their condition. A research assistant remained during deliberations to monitor jurors and their progress. After that, they completed the post-deliberation questionnaire, were debriefed, and finally, were compensated.

### 3.2. Analytic Plan

As in Experiment 1, confirmatory factor analyses were used for positive and negative affect (Table A3), perceptions of the defendant and his case, and perceptions of the plaintiff and her case (Table A4). Exploratory factor analysis was used to determine if we could reduce the perceptions of the deliberation process into meaningful factors. We separated perceptions of the deliberation process into verdict-related perceptions (e.g., “how much do you agree with the group verdict”) and damages-related perceptions (e.g., “how much do you agree with the group’s award amount”) because participants only answered the damages-related perceptions questions when they found for the plaintiff.

First, we used parallel analyses to determine that five factors (three verdict-related factors and two damages-related factors) should be retained (Horn, 1965; Sakaluk & Short, 2017). Then, we extracted factors relying on a maximum likelihood factoring extraction and an oblimin rotation (Table A5). We used standardized factor scores in subsequent analyses. Again, using these composite scores, difference scores were created for perceptions of the two litigants (perceptions of the plaintiff—perceptions of the defendant) and perceptions of the plaintiff’s and defendant’s cases (perceptions of the plaintiff’s case—perceptions of the defendant’s case). These difference scores were used in subsequent analyses.

#### 3.2.1. Liability Determination

*Pre-deliberation Juror Decision.* Across all conditions, 31.82% of participants found for the plaintiff. Unlike in Experiment 1, participants were given the option of answering that they were uncertain prior to deliberation. Thus, we conducted ordinal regressions to determine whether plaintiff ethnicity and verdict procedure predicted verdict.

*Post-deliberation Jury Decision.* Given that there were only 49 juries, we did not examine the interaction between plaintiff ethnicity and verdict procedure at the jury level. Instead, we present descriptive statistics and conduct a Fisher’s Exact Test to examine whether verdict procedure influenced jury decisions.

*Decision Changes.* We conducted an exploratory analysis examining whether plaintiff ethnicity and verdict procedure predicted changes in the likelihood of participants changing their verdict from either liable to not liable or vice versa during deliberation. We operationalized participants’ change in verdict by subtracting their pre-deliberation verdict from their deliberation verdict. For this analysis, we did not include participants who reported being uncertain (pre-deliberation) or juries that were hung (during deliberation). Thus, participants who did not change their verdict during deliberation scored a zero, participants who moved from liable to not liable scored a negative one, and participants who moved from not liable to liable scored a one.

#### 3.2.2. Damage Awards

*Pre-deliberation Juror Awards.* Although participants were only supposed to provide damages if they found for the plaintiff, many participants awarded damages even if they reported being uncertain. Therefore, for the following analyses, we examined the damage awards among the 119 (54.09%) jurors who either found for the plaintiff (*n* = 70, 33.82%) or indicated they were uncertain (*n* = 49, 22.27%). Across all conditions, damages ranged from USD 0 to USD 1,000,000, *M* = USD 47,628.14, *SD* = USD 131,544, *Median* = 10,000, *IQR* = 37,500. Eight mock jurors awarded zero dollars, despite finding for the plaintiff. Again, the damage awards were positively skewed, and we conducted regressions assuming a gamma distribution, adding a constant of one to each value to account for zeros.

*Post-deliberation Jury Awards.* Juries only provided damages if they found for the plaintiff. Therefore, we only examined jury-level damages from juries that found for the plaintiff. As only seven juries awarded damages, we did not conduct inferential statistics on jury-level damage awards. Instead, we only report descriptive statistics.

#### 3.2.3. Perceptions of the Parties

We examined whether perceptions of the parties changed depending on plaintiff ethnicity and verdict procedure. We conducted two-way ANOVAs examining whether the difference between perceptions of the plaintiff and defendant, and the difference between the plaintiff’s case and the defendant’s case, varied based on plaintiff ethnicity, verdict procedure, and the interaction of the two. In other words, we used the difference scores created by subtracting the perception of the defendant from the perception of the plaintiff, and the perception of the defendant’s case from the perception of the plaintiff’s case, as dependent variables and plaintiff ethnicity, verdict procedure, and the interaction of the two as independent variables.

#### 3.2.4. Perceptions of the Process

Finally, we examined whether perceptions of the process changed depending on plaintiff ethnicity and verdict procedure. We conducted five two-way ANOVAs examining whether effort in trial, power in deliberation, confidence in verdict, method of determining damages, and confidence in damage award differ based by plaintiff ethnicity, verdict procedure, and the interaction of the two.

### 3.3. Results

#### 3.3.1. Liability Determination

**Figure 3:**
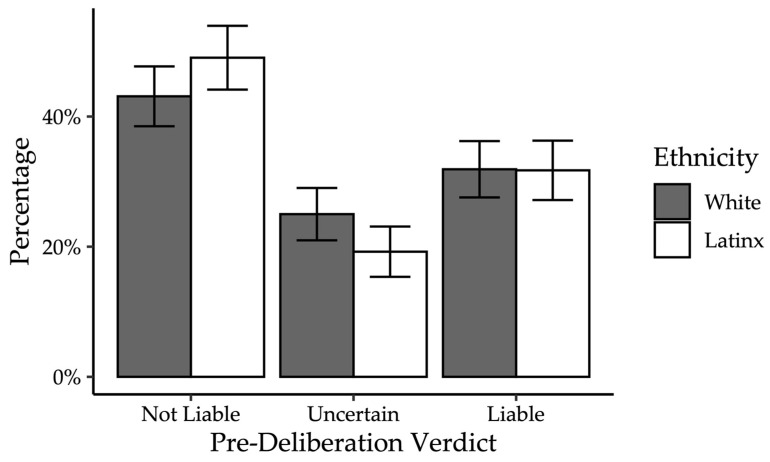
Percentage of Mock Jurors Who Gave each Verdict Across Plaintiff Ethnicity Conditions.

*Pre-deliberation Juror Decisions.* Contrary to expectations (Hypothesis 1), plaintiff ethnicity did not significantly predict verdicts, *b* = −0.14, *SE* = 0.25, Wald = −0.33, *p* = 0.57, *OR* = 0.87. These results did not change when we excluded uncertain jurors, *b* = −0.13, *SE* = 0.31, Wald = −0.19, *p* = 0.66, *OR* = 0.89; see Figure 3. We also examined verdict confidence. Again, contrary to expectations (Hypothesis 1), plaintiff ethnicity did not significantly predict verdict confidence, *F*(1, 218) = 0.47, *p* = 0.49, *ηp*^2^ = 0.002.

As with Experiment 1 and in alignment with Hypothesis 3 and Wiggins and Breckler (1990), mock jurors’ perceptions of the plaintiff’s and defendant’s cases independently predicted their pre-deliberation verdict, *b* = 0.88, *SE* = 0.11, *p* < 0.001, *OR* = 2.42. However, unlike Experiment 1, participants’ perceptions of the plaintiff and defendant also predicted mock juror pre-deliberation verdict, *b* = 0.90, *SE* = 0.11, *p* < 0.001, odds ratio = 2.47. This is consistent with the findings from Wiggins and Breckler (1990). As in Experiment 1, positive and negative affect were not significant predictors of verdict, *p*s > 0.27.

*Post-deliberation Jury Decision.* Descriptively, before deliberation, 47.97% of mock jurors in the special verdict with reason-giving verdict condition voted not liable. After deliberation, 92.59% of mock juries in the special verdict with reason-giving condition voted not liable. In contrast, 43.30% of mock jurors in the general verdict condition voted not liable, compared to 40.91% of mock juries. In other words, when mock juries deliberated using the general verdict procedure, the proportion of not-liable verdicts was relatively consistent with jurors’ pre-deliberation verdicts. However, when mock juries deliberated using the special verdict with reason-giving procedure, they were descriptively much more likely to return a verdict of not liable, relative to their pre-deliberation verdicts. Table 4 shows the percentage of each verdict (liable, hung, or not liable) for each level of plaintiff ethnicity and verdict procedure. We conducted a Fisher’s Exact Test for verdict collapsing across plaintiff ethnicity, given there was no evidence of an effect of ethnicity pre-deliberation. Hung juries were omitted from analysis given that juries rendering a decision using the special verdict with reason-giving procedure could not be hung, as there was no unanimity requirement. Results indicate a significant effect of verdict procedure, *p* = 0.04, *OR* = 0.15, 95% *CI* [0.01, 1.13]. As shown in Figure 3, mock juries were significantly less likely to find for the plaintiff in the special verdict with reason-giving procedure compared to when rendering a general verdict.

*Decision Changes.* Contrary to expectations (Hypothesis 1), plaintiff ethnicity did not predict change in verdict pre- to post-deliberation, *F*(1, 134) = 1.07, *p* = 0.30, *ηp*^2^ = 0.008. However, there was a significant main effect of verdict procedure such that participants were significantly more likely to change their pre-deliberation verdict from liable to not liable post-deliberation when using the special verdict with reason-giving procedure (*M* = −0.30, *SE* = 0.05) than the general verdict procedure (*M* = −0.08, *SE* = 0.07, *F*(1, 134) = 4.78, *p* = 0.03, *ηp*^2^ = 0.03), suggesting special verdicts with reason-giving led to more favorable outcomes for the defendant. There was no significant interaction between plaintiff ethnicity and verdict procedure (Hypothesis 3), *F*(1, 134) = 1.72, *p* = 0.19, *ηp*^2^ = 0.01.

#### 3.3.2. Damage Awards

**Figure 4:**
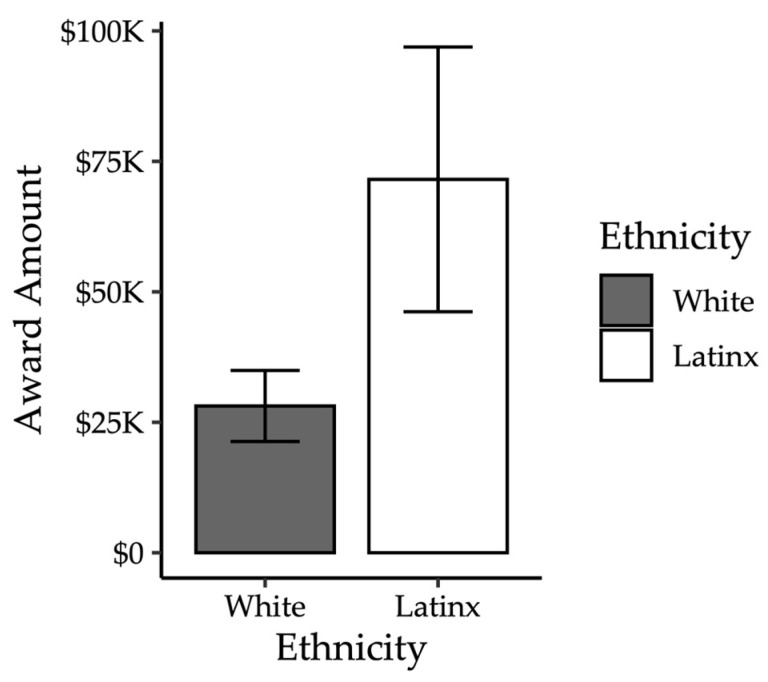
Average Damages Awarded Across Plaintiff Ethnicity Conditions.

*Pre-Deliberation Juror Awards.* Next, we examined the impact of plaintiff ethnicity on pre-deliberation damage awards. Unlike Experiment 1, analyses revealed that the overall model was significant, χ^2^ = 25.34, *p* = 0.03. Contrary to hypotheses, participants in the Latinx plaintiff condition awarded significantly more in damages than participants in the non-Latinx plaintiff condition, *b* = 0.93, *SE* = 0.42, *p* = 0.03, exp(*b*) = 2.54, as shown in Figure 4. We also examined whether these results differed based on participant verdict (liable or hung). There was no interaction between plaintiff ethnicity and participant verdict, *b* = 0.88, *SE* = 0.81, *p* = 0.28, exp(*b*) = 2.41.

*Post-deliberation Jury Awards.* As with juror-level decisions, juries only provided damages if they found for the plaintiff (*n* = 7; 14.29%). Therefore, we only examined jury-level damages from juries that found for the plaintiff. As only seven juries awarded damages, we did not conduct inferential statistics on jury-level damage awards. Instead, we only report descriptive statistics (Table 5).

#### 3.3.3. Perceptions of the Parties

**Figure 5:**
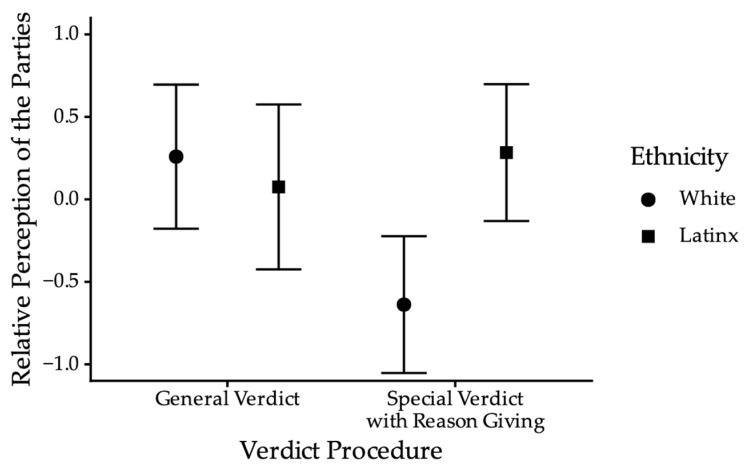
Relative Perceptions of the Parties. *Note*. Positive numbers indicate that the plaintiff was viewed more favorably than the defendant, whereas negative numbers indicate that the defendant was viewed more favorably than the plaintiff.

As shown in Table 6, there was a significant interaction between plaintiff ethnicity and verdict procedure on perceptions of the party. As shown in Figure 5, in the non-Latinx plaintiff condition, participants viewed the plaintiff more positively when using the general verdict procedure, *M* = 0.26, and the defendant more positively in the special verdict with reason-giving condition, *M* = −0.64, *t*(215) = 2.94, *p* = 0.004, *d* = 0.40. In the Latinx plaintiff condition, there was no difference between using the general, *M* = 0.08, and special verdict with reason-giving procedures, *M* = 0.28, *t*(215) = −0.63, *p* = 0.53, *d* = −0.09. There was also a main effect of plaintiff ethnicity on perceptions of the case. However, although the overall main effect was significant, pairwise comparisons suggest that, after correcting for multiple comparisons, the effect is only marginal. Participants viewed the plaintiff’s case as weaker than the defendant’s case in the non-Latinx plaintiff condition, *M* = −0.22, and the plaintiff’s case as stronger than the defendant’s case in the Latinx plaintiff condition, *M* = 0.22, *t*(215) = −1.90, *p* = 0.059, *d* = 0.26. No other main effects or interactions were significant.

#### 3.3.4. Perceptions of the Process

As shown in Table 7, there was a significant effect of verdict procedure on confidence in the verdict. Participants reported increased confidence in their group verdict when using the special verdict with reason-giving procedure (*M* = 0.17, *SE* = 0.08) compared to the general verdict procedure (*M* = −0.21, *SE* = 0.09), *t*(216) = −3.13, *p* = 0.002, *d* = 0.43. No other main effects or interactions were significant.

### 3.4. Discussion

Experiment 2 examined the potentially interactive effects of plaintiff ethnicity and verdict procedure, comparing the general verdict and the special verdict with reason-giving utilizing more ecologically valid measures and a more diverse sample compared to Experiment 1.

Despite changes from the experimental manipulation of race in Experiment 1 to the manipulation of ethnicity in Experiment 2, no support was found for Hypothesis 1: plaintiff ethnicity was again not predictive of verdict. In contrast to Experiment 1, in Experiment 2, plaintiff ethnicity was predictive of damages such that mock jurors awarded more damages for the Latinx plaintiff compared to the non-Latinx plaintiff pre-deliberation. One possibility is that participants responded in a socially desirable way in order to avoid presenting themselves as racist (Hunt, 2015; Salerno et al., 2023). In contrast, it is possible that participants who found for the Latinx plaintiff perceived that the defamation was caused in part by the defendant’s own biases/racism and, in turn, perceived the defamatory statements as an additional form of discrimination. That is, when participants have more discretion (as they do in damage award decisions, compared to verdict decisions), they might be more influenced by external influences, such as moral outrage at the defendant’s perceived racism (Thulin & Bicchieri, 2016).

Though no inferential statistical tests could be conducted on juries’ post-deliberation damage awards because of the low number of jury damage awards, interesting patterns emerged. Overall, the greatest damages were awarded to the Latinx plaintiff by juries who used the special verdict with reason-giving procedures. In contrast, for juries using the special verdict with reason-giving procedures, *no damages* were awarded for the non-Latinx plaintiff. In stark contrast, for juries utilizing general verdict procedures, damages were higher for the non-Latinx plaintiff.

Paralleling findings from Experiment 1 regarding special verdicts. In Experiment 2, we found support for Hypothesis 2: Juries rendering a verdict utilizing a special verdict with reason-giving procedure were more likely to find for the defendant compared to those utilizing a general verdict procedure. Exploratory analyses were conducted to examine changes in verdict preference for jurors (pre-deliberation) in comparison to juries’ decisions (post-deliberation). For jurors assigned to the general verdict procedures, their pre- and post-deliberation verdict decisions were nearly identical (43.30% and 40.91%, respectively). However, for jurors assigned to the special verdict with reason-giving procedures, a significant number of jurors who found for the plaintiff pre-deliberation (47.97%) ultimately found the defendant to be not liable following deliberation (92.59%). Mock jurors’ pre-deliberation verdicts represent their unstructured, holistic perceptions of the evidence and of their leading verdict preference, paralleling the process of a general verdict. In contrast, their post-deliberation verdicts having been structured by the special verdict with reason-giving likely reduced juries’ abilities to rely on intuitive, gut-based approaches to rendering a verdict.

In support of Hypothesis 3, and in line with findings from Experiment 1, mock jurors’ perceptions of the plaintiff’s and defendant’s cases predicted verdict. However, unlike Experiment 1, participants’ perceptions of the plaintiff and defendant, irrespective of perceptions of their cases, also predicted verdict. Further, following deliberation, plaintiff ethnicity and verdict procedure interacted to predict perceptions of the parties. Specifically, mock jurors in the non-Latinx conditions perceived the plaintiff’s case to be stronger, and the defendant’s case to be weaker, when utilizing general compared to special verdict with reason-giving procedures. However, in the Latinx plaintiff conditions, perceptions of the party did not differ across verdict procedures.

Exploratory analyses were conducted to examine mock jurors’ perceptions of the deliberation processes to examine whether such perceptions varied by verdict procedure. Jurors’ perceptions of the deliberation processes did not differ by verdict procedure when considering perceptions of power during deliberation, the methods they used when determining damages, or their confidence in the damages they awarded. However, jurors expressed significantly more confidence in their verdict decisions when utilizing special verdict with reason-giving versus general verdict procedures.

## 4. General Discussion

Across two experiments, we tested whether plaintiff race (Experiment 1) or plaintiff ethnicity (Experiment 2) influenced mock juror perceptions of a plaintiff, and whether various verdict procedures (Experiment 1, general, special, general verdicts with reason-giving either before or after rendering a verdict; Experiment 2, general verdict, special verdict with reason-giving) might reduce any influence of bias on jurors’ and juries’ decisions.

In both experiments, no evidence was found that either the plaintiff’s race or ethnicity influenced juror or jury verdicts, regardless of verdict procedure used. Though real-world data regarding civil juries suggests that juries award more to White and male plaintiffs, compared to plaintiffs of color and female plaintiffs (Girvan & Marek, 2016), recent research suggests that laboratory psycholegal research often produces counterintuitive race effects that are inconsistent with real-world data (Smalarz et al., 2023).

In contrast to prior research finding that jurors’ verdicts are similar when utilizing general or special verdicts (e.g., Wiggins & Breckler, 1990), across two experiments, we found that jurors and juries were significantly less likely to find for the plaintiff when using either a special verdict or special verdict with reason-giving compared to those using general verdict procedures. Further, a significant number of jurors who found for the plaintiff pre-deliberation ultimately found the defendant to be not liable following deliberation.

These findings are important, as special verdict and special verdict with reason-giving procedures take away jurors’ and juries’ power to determine the ultimate verdict: When utilizing the special verdict or special verdict with reason-giving procedures, jurors and juries had no opportunity to make a holistic decision regarding the defendant’s liability, as liability was determined by jurors’ responses to the four factual questions regarding the case. Special verdicts, which require jurors to respond to questions of fact (Fed. R. Civ. P. 49(b)(1), n.d.; Nepveu, 2003), may not allow them to rely on their previously constructed, holistic narratives of the case evidence. Further, jurors may not appreciate the implications of their responses to these answers of fact, or how they might relate to ultimate liability (Iuliano, 2014). Indeed, some scholars argue that informing jurors of the implications of their responses to special verdict questions will influence their answers (Schaffer, 1981).

The current study provides some evidence to suggest that when jurors rendered a general verdict, they distorted incoming information to match their intuitive beliefs about liability in this case. This finding is supported by other research finding that individuals distort incoming information to conform to an existing preference (e.g., Russo et al., 1996). Mock jurors in the special verdict conditions, including those who rendered a verdict using a special verdict or special verdict with reason-giving, who could not make an ultimate determination of liability were significantly more likely to find for the defendant. In the current case, mock jurors were likely unaware of the ramifications of answering the four factual questions on the special verdict forms, either when rendering a verdict using a special verdict or special verdict with reason-giving. The process of answering individual questions may have disrupted the common tendency to decide the case holistically (Henderson et al., 1995). A comparison of pre- versus post-deliberation liability determination provides further evidence: Before exposure to any verdict form, 45.9% of mock jurors found the defendant not liable. Following deliberation and exposure to the verdict procedures, 40.9% of mock jurors rendering a general verdict found the defendant not liable, in comparison to 92.6% of mock jurors rendering a special verdict with reason-giving.

These findings contrast with other research suggesting that jurors’ utilizing general or special verdicts decide cases similarly (Wiggins & Breckler, 1990), but in line with Sood (in press) who found that the impact of general verdicts versus general verdicts with written questions depended on the case. It is of note that our special verdict procedure (Experiment 1) and modified special verdict procedure with reason-giving (Experiment 2) differ from those used by Sood (in press). The current experimental procedures mirror the verdict procedure used by Wiggins and Breckler (1990) in Experiment 1 (i.e., a special verdict) but employed a different procedure than theirs in Experiment 2 (i.e., a modified special verdict procedure that included reason-giving). Further, a few case-specific differences exist between the current research and work by Wiggins and Breckler (1990). For instance, mock jurors in the current research heard only one claim of defamation, while those in the research conducted by Wiggins and Breckler (1990) heard two claims. Further, Wiggins and Breckler (1990) argue “special verdicts are more likely to be biased against one of the parties, relative to general verdicts, when the jury does not know the legal consequences of answers to special verdict questions” (p. 36). It is possible that jurors in the current study who rendered a special verdict were not aware of the legal implications of their answers to the factual questions: Mock jurors were significantly more likely in the other three conditions to find for the plaintiff, whereas mock jurors who rendered a special verdict were much more likely to find for the defendant, in line with Sood’s recent research (in press). This makes intuitive sense if in fact mock jurors were unaware of the legal implications of their answers to the fact-based questions: if so, the special verdict procedure, with or without reason-giving, may have prevented them from rendering a decision based on their holistic view of the case.

In addition, in Wiggins and Breckler’s (1990) study, the addition of a second claim of defamation might have had an additive impact such that mock jurors, aware of the legal implications of the special verdict form, with or without reason-giving, were equally likely to find for the plaintiff as those in the general verdict condition. Wiggins and Breckler (1990, p. 32) argue that their case materials may have produced equal bias against the plaintiff as defendant: “the number of special verdict questions could have produced a bias against the plaintiff, whereas the emphasis placed on multiple legal claims may have produced a bias against the defendant.”

Contrary to other research (Heuer & Penrod, 1994; Wiggins & Breckler, 1990), in Experiment 1, verdict confidence did not vary across conditions. In contrast, in Experiment 2, significant differences arose regarding juror confidence: mock jurors were significantly more confident in their verdict preference when utilizing special verdict with reason-giving compared to general verdict procedures. An important difference between experiments 1 and 2 was the inclusion of deliberation procedures in Experiment 2. Jurors in Experiment 2 likely felt more confident in their verdicts generally, but importantly, they were most confident when rendering a collective verdict using the more structured procedures associated with special verdicts with reason-giving.

Research in other domains (i.e., education) suggests that task-induced involvement load is predictive of confidence in related tasks (Teng, 2017). As this finding may relate to special and special verdicts with reason-giving, such structured procedures likely induce more task-related load compared to the holistic judgment of a general verdict. In turn, this induced task load likely improved jurors’ confidence in their judgments for the special verdict with reason-giving procedures when deliberation was present.

### 4.1. Legal Implications

Across two experiments, mock jurors and juries were significantly less likely to find for the plaintiff, regardless of plaintiff race or ethnicity, when rendering a special verdict or special verdict with reason-giving, as compared to a general verdict. Because jurors may not comprehend the implications of their responses to questions of fact and how they relate to ultimate liability determination (Iuliano, 2014), special verdicts and special verdicts with reason-giving arguably reduce juries’ power. Some scholars even argue that informing jurors of the implications of their responses to special verdict questions will influence their answers, perhaps in line with preexisting biases (e.g., Schaffer, 1981). Importantly, the cases used across both experiments were rather ambiguous in nature: thus, we might expect mock jurors and juries to find for the plaintiff and defendant roughly 50% of the time, which stands in stark contrast to our findings regarding special verdicts and special verdicts with reason-giving. This may suggest that special verdicts and special verdicts with reason-giving reduce fairness for the plaintiff’s cases and reduce the jury’s power to determine the ultimate verdict in line with their preferences.

Determining the strength of various verdict procedures is both an empirical and normative issue: While in some cases it might be possible to establish which verdict procedure is strongest in terms of juror decision making (e.g., if empirical research discovered that one procedure was most likely to reduce juror bias), there are several other important values that may be met by a variety of these procedures (Z. Clopton, personal communication, 29 September 2017). For instance, special or reasoned verdicts could help promote transparency in juror decision making and could help parties on appeal. Such transparency could also improve the public’s perception of jury decision making, which could promote a sense of legitimacy for the institution (Grimmelikhuijsen & Klijn, 2015; Trinkner et al., 2018).

However, special verdicts, special verdicts with reason-giving, and the sorts of reasoned verdict procedures used abroad suffer from a lack of procedural standardization, which could also reduce trial efficiency. Further, some legal scholars argue that general verdicts allow jurors the most freedom to deliberate and make jurors less vulnerable to external influence (i.e., as in abroad, where judges review reasoned verdicts and may make suggestions for changes; Burd & Hans, 2018). In the event that future research finds evidence to suggest that reasoned verdicts promote juror decision making, procedures may remain unchanged so as to preserve jury independence (Abramson, 2015) or to preserve other competing values (e.g., freedom from external influences, freedom of deliberation; Hans & Jolivet, 2016).

### 4.2. Limitations and Future Directions

Future research should explore the impact of these varied verdict procedures across a myriad of case types, including both criminal and civil trials. Although special verdict procedures, with or without reasoning giving, are not currently employed in the guilt phase of criminal cases, penalty phase verdict forms in capital cases often mirror special verdict procedures (i.e., requiring juries to answer a series of fact-based questions before recommending the sentence; *Penalty Phase Verdict Forms*, 2025) and even in non-capital cases (e.g., where a jury must find evidence of aggravating factors or statutory sentence enhancements under *Apprendi v. New Jersey* (2000); see, for example 11A Wash. Prac., Pattern Jury Instr. Crim. WPIC 190.02, n.d.; ICJI 1024 DWP Special Verdict Instruction—Enhancement, n.d.). It is important to assess the likelihood that a reasoning requirement might reduce juror bias; thus, future studies should include a stronger race/ethnicity induction or other biasing information. In addition, to investigate further the possibility that the reasoning requirement may mitigate the impact of strong moral and/or emotional intuitions, in criminal cases, severe crimes should be presented, and within civil cases, torts that include physical and psychological harm.

Experiment 2 strengthened the ecological validity of Experiment 1 in several important ways. However, it is important to note that Experiment 2 was conducted during the height of the COVID-19 pandemic using Zoom. As such, the process of deliberation was made more difficult, and was less controlled, than would be an in-laboratory, in-person deliberation paradigm.

As discussed above, in the current research, mock jurors were likely unaware of the ramifications of answering the factual questions on the special verdict forms, whether or not reasons were required. Future research using similar procedures could ask jurors to reflect on their answers to a special verdict form and to report what verdict they believe has been rendered.

## 5. Conclusions

Special and reasoned verdicts have the potential to improve transparency to the parties and to the general public. However, they also interfere with the jury’s traditional independence in decision making. The lack of standardization in real-world verdict procedures with a reasoning requirement makes it difficult to assess the effects of these procedures, including whether special and reasoned verdicts reduce arbitrary decision making. The two experimental studies reported here show that the verdict procedures can change the final verdicts, and those who wish to use alternatives to general verdicts should proceed with caution. More research is needed to further explore the impact of a reasoning requirement on juror decision making.

## Figures and Tables

**Table 1:** Sample Characteristics.

	Total Sample (*N* = 267)		Convenience Sample (*n* = 160)		MTurk Sample (*n* = 107)		
	*n* or Range	% or Mean	*n* or Range	% or Mean	*n* or Range	% or Mean	*p* Value
*Age*	19–77	38.03	19–77	40.72	22–66	34.13	<0.001
*Race/Ethnicity*							<0.001
Black	24	9.1%	5	3.2%	19	17.8%	
Latinx	19	7.2%	13	8.3%	6	5.6%	
Asian	10	3.8%	2	1.3%	8	7.5%	
Native American	2	0.8%	1	0.6%	1	0.9%	
White	200	76%	130	83.3%	70	65.4%	
Other	8	3.0%	5	3.2%	3	2.8%	
*Sex/Gender Identification*							<0.001
Cis-Male	92	35.4%	34	22.2%	58	54.2%	
Cis-Female	147	56.5%	107	69.9%	40	37.4%	
Trans-Male	16	6.2%	9	5.9%	7	6.5%	
Trans-Female	1	0.4%	0	0.0%	1	0.9%	
Non-binary	1	0.4%	1	0.7%	0	0.0%	
Other	3	1.2%	2	1.3%	1	0.9%	
*Education*							<0.01
<High School	1	0.4%	0	0.0%	1	0.9%	
High School/GED	20	7.6%	10	6.4%	10	9.3%	
Some College	72	27.4%	42	26.9%	30	28.0%	
College Graduate	85	32.3%	39	25.0%	46	43.0%	
Some Graduate School	25	9.5%	20	12.8%	5	4.7%	
Graduate Degree	60	22.8%	45	28.8%	15	14.0%	
*Verdict for Plaintiff*	176	66.2%	105	66.0%	71	66.4%	0.96

*Note.* *p*-value indicates the significance of the difference between the convenience sample and the MTurk sample on the associated variable.

**Table 2:** Special Verdict Broken Down by Question.

Defamatory Statement (Q1)	Injured the Plaintiff (Q2)	Malice or Reckless Disregard (Q3)	The Statement Was True (Q4)	*n (%)*
Yes	Yes	Yes	No	27 (38.03)
No	No	No	No	16 (22.54)
No	No	No	Yes	3 (4.23)
No	Yes	No	No	3 (4.23)
No	Yes	Yes	No	1 (1.41)
Yes	No	No	No	4 (5.63)
Yes	Yes	No	No	8 (11.27)
Yes	Yes	No	Yes	2 (2.82)
Yes	Yes	Yes	Yes	6 (8.45)
Yes	Yes	Yes	Not Answered	1 (1.41)

*Note.* Only the first row (three yeses and one no) resulted in a liable verdict.

**Table 3:** Sample Characteristics.

	Total Sample (*N* = 220)		SONA Sample (*n* = 116)		Craigslist Sample (*n* = 104)		
	*n* or Range	% or Mean	*n* or Range	% or Mean	*n* or Range	% or Mean	*p* Value
*Age*	18–82	27.21	18–55	21.12	18–82	33.88	<0.001
*Race/Ethnicity*							<0.001
Black	22	10.0%	1	0.9%	21	20.2%	
Latinx	13	5.9%	6	5.2%	7	6.7%	
Asian	6	2.7%	2	1.7%	4	3.9%	
Native American	3	1.4%	3	2.6	—	—	
Pacific Islander	3	1.4%	—	—	3	2.9%	
White	168	76.4%	103	88.8%	65	62.5%	
Other	5	2.3%	1	0.9%	4	3.9%	
*Sex/Gender Identification*							0.28
Cis-Male	79	35.9%	46	39.7%	33	31.7%	
Cis-Female	140	63.6%	69	59.5%	71	68.3%	
Trans-Male	1	0.5%	1	0.9%	—	—	
*Education*							<0.001
<High School	1	0.5%	1	0.9%	—	—	
High School/GED	26	11.8%	22	19.0%	4	3.9%	
Some College	123	55.9%	86	74.1%	37	35.6%	
College Graduate	34	15.5%	6	5.2%	28	26.9%	
Some Graduate School	9	4.1%	—	—	9	8.7%	
Graduate Degree	23	10.5%	—	—	23	22.1%	
Other	3	1.4%	1	0.86	2	1.9%	
Prefer not to answer	1	0.5%	—	—	1	1.0%	
*Verdict for Plaintiff*	101	45.9%	60	51.7%	41	39.42%	0.09

*Note.* *p*-value indicates the significance of the difference between the SONA sample and the Craigslist sample on the associated variable.

**Table 4:** Jury-Level Verdicts.

Plaintiff Ethnicity	Non-Latinx		Latinx	
	General Verdict	Special Verdict with Reason-Giving	General Verdict	Special Verdict with Reason-Giving
Liable	3 (25.00%)	0 (0.00%)	2 (20.00%)	2 (16.67%)
Hung	4 (33.33%)	N/A	4 (40.00%)	N/A
Not Liable	5 (41.67%)	15 (100%)	4 (40.00%)	10 (83.33%)

*Note.* Due to the nature of the special verdict with reason-giving procedure, which allows for a majority vote rather than requiring unanimity, juries cannot be hung.

**Table 5:** Jury-Level Damage Awards.

Plaintiff Ethnicity	Non-Latinx			Latinx		
	Range	Mean (*SD*)	Median (*IQR*)	Range	Mean (*SD*)	Median (*IQR*)
General Verdict	35,000, 160,000	88,333.33 (64,485.14)	70,000.00 (62,500.00)	22,400, 40,000	31,200.00 (12,445.08)	31,200.00 (13,046.88)
Special Verdict with Reason-giving	—	—	—	12,950, 510,000	261,475.00 (351,467.43)	261,475.00 (248,525.00)

*Note.* No juries utilizing the special verdict with reason-giving procedure found the defendant liable in the non-Latinx plaintiff condition; as such, they did not determine damages for the non-Latinx plaintiff.

**Table 6:** The Impact of Plaintiff Ethnicity and Verdict Procedure on Relative Perceptions of the Parties and the Case.

	*F*	*DF*	*p*	*ηp* ^2^
	Perception of the Parties			
Plaintiff Ethnicity	3.42	(1, 215)	0.07	0.02
Verdict Procedure	2.97	(1, 215)	0.09	0.01
**Ethnicity × Procedure**	**6.04**	**(1, 215)**	**0.01**	**0.03**
	Perception of the Case			
**Plaintiff Ethnicity**	**4.16**	**(1, 215)**	**0.04**	**0.02**
Verdict Procedure	2.02	(1, 215)	0.16	0.009
Ethnicity × Procedure	2.98	(1, 215)	0.09	0.01

*Note.* *p* < 0.05 is in **bold**.

**Table 7:** The Impact of Plaintiff Ethnicity and Verdict Procedure on Perceptions of the Process.

	*F*	*DF*	*p*	*ηp* ^2^
	Effort in Trial			
Plaintiff Ethnicity	0.007	(1, 216)	0.93	<0.001
Verdict Procedure	2.13	(1, 216)	0.15	0.01
Ethnicity × Procedure	0.002	(1, 216)	0.96	<0.001
	Power in Deliberation			
Plaintiff Ethnicity	0.01	(1, 216)	0.91	<0.001
Verdict Procedure	3.27	(1, 216)	0.07	0.01
Ethnicity × Procedure	0.74	(1, 216)	0.39	0.003
	Confidence in Verdict			
Plaintiff Ethnicity	1.14	(1, 216)	0.29	0.005
**Verdict Procedure**	**10.24**	**(1, 216)**	**0.002**	**0.05**
Ethnicity × Procedure	0.88	(1, 216)	0.35	0.004
	Method of Determining Damages			
Plaintiff Ethnicity	1.30	(1, 119)	0.26	0.01
Verdict Procedure	3.38	(1, 119)	0.07	0.03
Ethnicity × Procedure	2.86	(1, 119)	0.09	0.02
	Confidence in Damage Award			
Plaintiff Ethnicity	0.27	(1, 119)	0.60	0.002
Verdict Procedure	3.64	(1, 119)	0.06	0.03
Ethnicity × Procedure	0.54	(1, 119)	0.46	0.005

*Note.* *p* < 0.05 is in **bold**.

## Data Availability

The original data presented in the studies are openly available at https://osf.io/m7hzj/?view_only=9b51773dfaee475aa586cf79b75c0d3b.

## Notes

1 All materials and measures are available at https://osf.io/m7hzj/overview?view_only=9b51773dfaee475aa586cf79b75c0d3b.

2 See note 1 above.

## Appendix A

**Table A1:** Confirmatory Factor Analysis (CFA) loadings and cumulative variance for affect in Experiment 1.

Item	Loading
**Negative Affect**	
Afraid	0.771
Anger	0.773
Anxious	0.740
Ashamed	0.855
Disgusted	0.751
Guilty	0.819
Hostile	0.742
Irritable	0.808
Jittery	0.791
Sad	0.785
Upset	0.739
Contemptuous	0.649
Average Variance Extracted (AVE): 58.71%	
**Positive Affect**	
Attentive	0.108
Calm	0.317
Enthusiastic	0.797
Happy	0.678
Inspired	0.782
Strong	0.693
Surprised	0.436
AVE: 35.78%	

**Table A2:** Confirmatory Factor Analysis (CFA) loadings and cumulative variance of perceptions of the plaintiff and defendant in Experiment 1.

Item	Loading
**Perceptions of the Defendant**	
Believable	0.843
Blameless	0.793
Candid	0.835
Honest	0.857
Likeable	0.813
Moral	0.817
Reputable	0.803
Right	0.790
Overall Impression	0.800
AVE: 66.87%	
**Perceptions of the Defendant’s Case**	
Believable	0.897
Convincing	0.937
Persuasive	0.868
Serious	0.660
Strong	0.848
AVE: 72.10%	
**Perceptions of the Plaintiff**	
Believable	0.865
Blameless	0.809
Candid	0.840
Honest	0.906
Likeable	0.885
Moral	0.894
Reputable	0.866
Right	0.891
Overall Impression	0.686
AVE: 73.35%	
**Perceptions of the Plaintiff’s Case**	
Believable	0.905
Convincing	0.944
Persuasive	0.846
Serious	0.740
Strong	0.869
AVE: 74.91%	

**Table A3:** Confirmatory Factor Analysis (CFA) loadings and cumulative variance for affect in Experiment 2.

Item	Loading
**Negative Affect**	
Afraid	0.603
Anger	0.765
Ashamed	0.74
Disgusted	0.432
Guilty	0.67
Hostile	0.589
Irritable	0.445
Jittery	0.459
Sad	0.598
Upset	0.617
AVE: 32.11%	
**Positive Affect**	
Attentive	0.411
Calm	0.42
Enthusiastic	0.823
Happy	0.732
Inspired	0.765
Strong	0.625
Surprised	0.350
AVE: 39.31%	

**Table A4:** Confirmatory Factor Analysis (CFA) loadings and cumulative variance of perceptions of the plaintiff and defendant in Experiment 2.

Item	Loading
**Perceptions of the Defendant**	
Believable	0.864
Blameless	0.772
Candid	0.808
Honest	0.856
Likeable	0.788
Moral	0.808
Reputable	0.827
Right	0.871
Overall Impression	0.855
AVE: 68.67%	
**Perceptions of the Defendant’s Case**	
Believable	0.871
Convincing	0.930
Persuasive	0.903
Serious	0.620
Strong	0.793
AVE: 69.99%	
**Perceptions of the Plaintiff**	
Believable	0.880
Blameless	0.826
Candid	0.852
Honest	0.895
Likeable	0.737
Moral	0.825
Reputable	0.848
Right	0.840
Overall Impression	0.762
AVE: 70.16%	
**Perceptions of the Plaintiff’s Case**	
Believable	0.819
Convincing	0.928
Persuasive	0.910
Serious	0.586
Strong	0.846
AVE: 70.35%	

**Table A5:** Exploratory Factor Analysis (EFA) loadings and cumulative variance of perceptions of the deliberation experience in Experiment 2.

Item	Loading
**Effort In Trial**	
Motivation to Reach a Verdict	0.923
Cognitive Effort in Reaching a Verdict	0.752
AVE: 21.3%	
**Power in Deliberation**	
Power in Deliberation	1.071
Power to Reach a Verdict	0.480
AVE: 42.4%	
**Confidence in Verdict**	
Agreement with Group Verdict	0.974
Perception that the Trial was Fair	0.525
Confidence in Group Verdict	0.446
AVE: 61.5%	
**Method of Determining Damages**	
Motivation to Determine Damages	0.710
Cognitive Effort in Determining Damages	0.729
Difficulty in Determining Damages	0.463
Role of Economic Damages	0.708
Role of Punishment	0.597
AVE: 30.7%	
**Confidence in Damage Award**	
Agreement with Group Damage Award	0.995
Confidence in Group Damage Award	0.910
AVE: 58.8%	
